# Effectiveness of training in evidence-based medicine skills for healthcare professionals: a systematic review

**DOI:** 10.1186/s12909-016-0616-2

**Published:** 2016-04-04

**Authors:** Lars Hecht, Susanne Buhse, Gabriele Meyer

**Affiliations:** University of Witten/Herdecke, Faculty of Health, School of Nursing Science, Witten/Herdeke, Germany; RED Institute for Medical Research and Education, Mühlenkamp 5, 23758 Oldenburg, Germany; University of Hamburg, Faculty of Mathematics, Informatics and Natural Sciences, Unit of Health Sciences and Education, Hamburg, Germany; Martin Luther University Halle-Wittenberg, Medical Faculty, Institute for Health and Nursing Science, Halle, Germany

**Keywords:** Evidence-Based Medicine, Evidence-Based Nursing, Complex intervention, Education, Health Personnel

## Abstract

**Background:**

Basic skills in evidence-based medicine (EbM) are indispensable for healthcare professionals to promote consumer-centred, evidence-based treatment. EbM training courses are complex interventions – a fact that has not been methodologically reflected by previous systematic reviews.

This review evaluates the effects of EbM training for healthcare professionals as well as the quality of reporting of such training interventions.

**Methods:**

We searched PubMed, EMBASE, CINAHL, Cochrane Library, ERIC, Campbell Library and PsycINFO up to 9/2014. Randomised controlled trials, controlled clinical trials as well as before-after trials were included. Authors were contacted in order to obtain missing data. Two independent reviewers extracted data and assessed risk of bias.

**Results:**

We reviewed 14.507 articles; *n* = 61 appeared potentially eligible; *n* = 13 involving 1,120 participants were included. EbM training shows some impact on knowledge and skills, whereas the impact on practical EbM application remains unclear. Risk of bias of included trials raises uncertainty about the effects. Description of complex interventions was poor.

**Conclusions:**

EbM training has some positive effects on knowledge and skills of healthcare professionals. Appropriate methods for development, piloting, evaluation, reporting and implementation of the training should be applied.

**Electronic supplementary material:**

The online version of this article (doi:10.1186/s12909-016-0616-2) contains supplementary material, which is available to authorized users.

## Background

Evidence-based medicine (EbM) is a prerequisite for decision-making in healthcare. All over the world, healthcare institutes follow the principles of EbM when reviewing and assessing the evidence for healthcare decision-making [1, 2]. The paradigm shift towards EbM challenges the methodological skills and attitude of healthcare professionals. A consumer-centred, evidence-based treatment requires basic EbM skills and scientific literacy [3, 4].

The transfer of evidence into routine care is often not optimal [5]. Barriers that impede the implementation of EbM have been extensively researched. Lack of time to put EbM into practice, false beliefs about EbM, insufficient support in the clinical setting and limited critical appraisal skills are the barriers that healthcare professionals most often face [6, 7].

There is empirical evidence that EbM training activities improve knowledge and skills needed for the critical appraisal of scientific papers [8]. Traditionally, training and continuing education in EbM focus on physicians. An early systematic review by Shaneyfeld and colleagues identified 104 trials on EbM training courses; *n* = 91 addressed physicians [9]. However, during the last few years curricula and textbooks aiming to achieve EbM competencies for allied healthcare professionals have been developed. Training courses in EbM skills for nurses, diabetes educators and other professionals associated with health have been shown to be feasible and well appreciated [10, 11]. Organizations like the Joanna Briggs Institute or the Centre of Evidence-Based Physiotherapy, which provide a variety of EbM workshops and learning opportunities for nurses, physical therapists, midwives, medical and allied health researchers, have been established. EbM resources have been made accessible, for instance, a free database of randomised trials, systematic reviews and clinical practice guidelines in physiotherapy. To address the increasing teaching demand and the need to improve the effectiveness of EbM, train-the-trainer courses have been developed [12]. Training in EbM for patients and consumer representatives is offered by some organisations [13, 14].

A variety of different approaches exists for teaching and learning EbM, for instance, by attending courses, conferences, workshops or journal clubs [15]. Recent systematic reviews showed inconsistent effects regarding the effectiveness of different EbM teaching and learning methods. Coomarasamy and Khan evaluated the effects of stand-alone versus clinically integrated teaching in EbM on several outcomes in postgraduates [16]. Stand-alone teaching was defined as classroom teaching, either didactic, interactive, or mixed. A total of 23 randomised controlled, controlled clinical as well as twelve before-after trials were included in this systematic review. Knowledge was assessed in 17 trials, critical appraisal skills in nine trials, changes in attitudes in six, and behavioural change in 14 trials. None of the trials evaluated clinical health outcomes. Stand-alone teaching as well as integrated teaching was effective in improving EbM knowledge but only clinically integrated teaching improved skills, attitudes, and behaviour [16]. Outcomes were predominantly determined by self-assessment. In contrast, a recently published Cochrane review on the effectiveness of training conducted to increase the “critical appraisal” skills only included trials if the assessment of outcome measures was based upon standardised and reliable instruments (e.g. tests, questionnaires). Three randomised controlled trials with 272 participants fulfilled the inclusion criteria. While a statistically significant improvement in participants’ critical appraisal knowledge was seen in two of the three trials, none of the three trials evaluated the process of care or patient outcomes [17].

Former systematic reviews dealing with EbM training activities have not taken the complexity of educational interventions into account. Complex interventions typically comprise interacting elements that are also influenced by contextual factors [18]. Educational interventions are often heterogeneous in their underlying theory, the methods used, the format and intensity, and the target population. The development of educational interventions requires great investment in testing procedures for their feasibility and acceptability prior to large-scale evaluations. The process of implementation should ideally be carefully prepared and piloted and it is recommended to take the whole chain of complex intervention development and evaluation into account while reviewing complex interventions. Thus, a non-customary approach for evidence synthesis is needed. The UK Medical Research Council (MRC) has provided the framework for understanding and appraising complex interventions [18–21]. All relevant patient outcome parameters need to be assessed, interdependencies between active elements should be taken into account, and all trials referring to development, evaluation and implementation of the educational intervention should be considered. The theoretical basis and its influence on the education program should be analysed. Adequate reporting of the elements and interdependencies within complex interventions are the prerequisite to interpret the outcomes.

To the best of our knowledge, a systematic review on EbM training for healthcare professionals with respect to the complexity of the educational interventions has not been performed. Therefore, the objective of this review is to evaluate the effects and the quality of the reporting regarding development and implementation of EbM training for healthcare professionals.

## Methods

The review protocol has been registered in PROSPERO International Prospective Register of Systematic Reviews (crd.york.ac.uk/prospero/index.asp Identifier CRD42014013579).

### Types of trials

We included all individual and cluster-randomised controlled trials (RCTs), controlled clinical trials (CCTs) as well as before-after trials. Non-blinded trials were included in the review, since blinding of participating healthcare professionals seemed to be unrealistic. The publication language was restricted to English and German.

### Types of participants

Healthcare professionals in any clinical or academic setting were included. Trials on EbM training solely for physicians, medical students, patients and patient representatives, managers or purchasers were excluded.

### Types of interventions

This review focuses on educational interventions aimed at improving EbM knowledge, skills, attitudes and behaviour in healthcare professionals. The interventions might cover the following contents: Formulating questions that could be answered by a systematic literature search; performing a systematic literature search; critically appraising selected publications; communicating trial results to consumer and patients.

We excluded trials that investigated the effects of teaching solely biostatistics or search strategies, programs focusing on specific health problems, medical education in general (not EbM in particular), and trials testing the effectiveness of implementing evidence-based guidelines.

### Types of outcome measures

1. Outcome measures were attitudes, knowledge, skills, and behaviour regarding EbM which were objectively assessed through validated instruments.
2. Impact of EbM training on the implementation of EbM in routine care and patient-relevant outcomes like mortality, morbidity, and quality of life were assessed.

### Search methods for identification of trials

The literature search strategy followed the Cochrane Handbook for Systematic Reviews of Interventions, version 5.1.0. [22]. MEDLINE, EMBASE, CINAHL, Cochrane Library, ERIC, Campbell Library and PsycINFO were searched systematically in September 2014.

The following terms were used: “Health Personnel (MeSH term)”, “dietician”, “dietitian”, “diabetes educator”, “evidence-based medicine”, “evidence-based nursing”, “evidence-based practice”, “evidence-based”, “journal club”, “critical read*”, “critical appraisal”, “science literacy”, “health literacy”, “risk literacy”, “education (MeSH term)”, “train*”

Reference lists of published reviews and included articles were checked for additional trials. If the full text was not available, the authors of the trials were contacted.

### Selection of trials

Two review authors (LH, SB) independently assessed titles and abstracts from the search. Eligible articles were assessed for inclusion. Disagreement was solved by consensus.

### Data extraction and management

Based upon CReDECI [23] and the CONSORT statement [24], we developed a standardized data extraction form that included information on the development, evaluation and implementation of complex interventions:

- Description of the intervention
- Description of the intervention’s development (e.g. theoretical and/or evidence base)
- Information on pilot testing
- Delivery of the intervention (who, how often, how long?)
- Description of the implementation strategy
- Description of any material and method used
- Method of assessing participants’ preferences/interests/experiences
- Description of process evaluation
- Information on costs/resources needed for the implementation of the intervention
- Description of what has been offered in the control group.

Data were extracted by two independent reviewers (LH, SB) and checked for accuracy. In case of discrepancy, the third review author (GM) was called in to reach consensus. Quality criteria following the Cochrane Handbook for Systematic Reviews of Interventions Version 5.1.0 [22] were applied in order to assess the risk of bias of included trials. Critical appraisal of trials was carried out by two independent reviewers. In the case of unclear or missing information, the corresponding author of the trial was contacted.

Since we found pronounced methodological heterogeneity, the trial results are presented in a narrative form only.

## Results

**Fig. 1:**
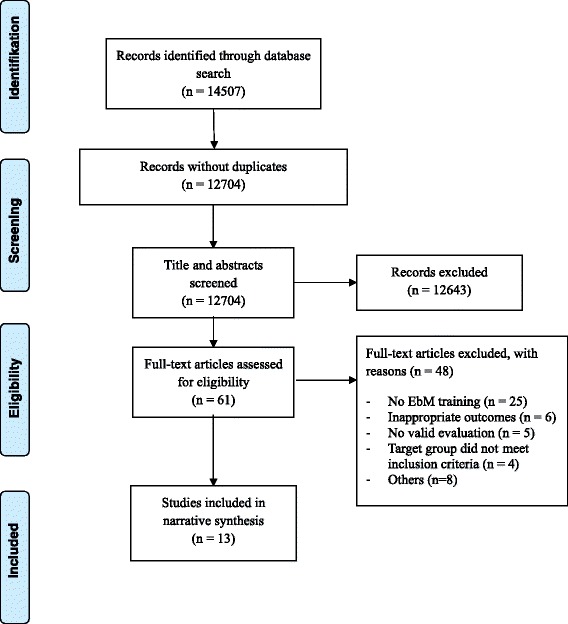
Flow diagram

A total of 14.507 articles were identified, of which 61 were considered for inclusion. After screening the full text articles, a total of 13 trials were included: four randomised controlled trials [25–28], two controlled clinical trials [29, 30] and seven before-after trials [11, 31–36]. Sample sizes ranged from *n* = 30 [26] to *n* = 168 [29] with a total of 1,120 participants. The reasons for exclusion are reported in the flow diagram (Fig. 1). In seven trials, participants’ age was not reported [11, 27, 29–31, 33, 34]. In the remaining trials, the age ranged from 18 to 64 years.

Trials were conducted in the United States of America [27, 30, 32, 35], the United Kingdom [26], Australia [31, 33, 34], Canada [36], Germany [11], Taiwan [29], Philippines [28] and Iran [25].

Healthcare professionals involved in these trials were predominantly nurses or nursing students, nurse managers, occupational therapists, physiotherapists, speech pathologists, dieticians and diabetes nurses or diabetes instructors with or without academic background and varying job experience.

The total trial duration ranged from eight weeks [35] to 27 months [32]. The characteristics of the included trials are summarized in Table 1.

**Table 1:** Characteristics of included studies (*n* = 12)

Authors	Design	Setting/Location	n (study completed)	Age (years)	Study/Observation Duration	Profession	Job Experience
Bennet et al.	Before-after trial	University of Queensland, Brisbane, Australia	94 (59)	Not reported	1 university semester course duration 13 weeks	Undergraduate final year occupational therapy students (*n* = 32) Postgraduate physiotherapy students (*n* = 27)	Students
Kim et al.	Before-after trial	Collaboration between hospitals and university, San Diego, California, USA	159 (111)	42 (range 22–64)	2008–2010 3 annual cohorts of nurses attending the 9-month collaborative regional fellowship program. Pre-test and post-test (after 9 months)	Staff nurse (*n* = 58) Nurse manager (*n* = 9) Clinical nurse specialist/Nurse educator/Nurse practitioner (*n* = 63)	16 (range: 1–42) years
Lizarondo et al.	Before-after trial	Healthcare facilities, Tasmania, Australia	93 (93)	Not reported	6 months	Speech pathologists (*n* = 10), physiotherapists (*n* = 19), social workers (*n* = 16), occupational therapists (*n* = 36), dieticians/nutritionists (*n* = 12)	Academic background: Undergraduate qualifications *n* = 53 Postgraduate qualifications *n* = 40 Length of clinical practice: <5 years *n* = 19 >5 but <10 years *n* = 17 >10 years *n* = 49 not reported *n* = 8
McCluskey et al.	Before-after trial	School of Exercise and Health Sciences, New South Wales, Australia	114 FU 1 (post intervention) 106 FU 2 (8 month post intervention) 51	Not reported	November 2001–March 2003	Professional occupational therapists	Qualification: Diploma *n* = 15/degree *n* = 99 Time since graduation: <5 years *n* = 29 >5 but < 10 years *n* = 19 >10 years *n* = 66
Meyer et al.	Before-after trial	Diabetes educator courses in Germany. Centres providing the diabetes educator graduate programme accredited by the German Diabetes Association	121 (93)	Not reported	2003–2004	Diabetes nurse specialists (65 %) or diabetes counsellors without a university degree, 3 % of the participants were dieticians with a university degree	Not reported
Varnell et al.	Before-after trial	Acute care setting Texas, USA	102 (98)	43.6 (range 22–62)	8 weeks	Registered nurses	14.7 (range 1–41) years
Yost et al.	Before-after trial	School of Nursing McMaster University, Hamilton, Canada	40 (21)	44.3 ± 9.2	May 2010–November 2010 (6 month)	Registered Nurse (*n* = 25) Advance practice nurse (*n* = 1) Physicians (*n* = 1) Librarian (*n* = 2) Other (*n* = 10) Not reported (*n* = 1) Main job function: Executive officer (*n* = 2) Associate medical officer of health (*n* = 1) Program manager/administrator (*n* = 14) Direct service/care provider (*n* = 5) Research (*n* = 2) Policy development/analysis (*n*= 1) Faculty (*n* = 8) Other (*n* = 6) Not reported *n* = 1	Baccalaureate (*n* = 20) Masters (*n* = 18) Other (*n* = 1) Not reported (*n* = 1)
Chen et al.	Nonrandomised controlled trial	2-year nursing program at one college, Taiwan	IG 94 (94) CG 74 (74)	Not reported	1 semester	Students graduated from junior nursing schools with clinical practicum experience	IG: (*n* = 13) Working experience (1 month to 2 years as a nurse) (*n* = 81) No working experience CG: (*n* = 6) Working experience (1 month to 2 years as a nurse) (*n* = 68) No working experience
Courey et al.	Nonrandomised controlled trial	University, Ohio	IG 19 (19) CG 39 (39)	Not reported	1 semester	First year students in a 2-year associate degree nursing program	Not reported
Jalali-Nia et al.	Randomised controlled trial	Baccalaureate nursing program, Teheran, Iran	IG 20 (20) CG 21 (21)	Not reported	1 semester	Students in the second year of the baccalaureate nursing program	Not reported
Stevenson et al.	Randomised controlled trial	Musculoskeletal physiotherapists working within the Community Trust North Staffordshire, UK	IG 17 (16) CG 13 (11)	18–29 IG = 1 CG = 0 30–49 IG = 8 CG = 10 ≥50 IG = 8 CG = 3	Six month	Physiotherapists of all grades	Average time since qualification: IG 25 years CG 23 years
Levin et al.	Randomised controlled trial	Home care setting/community health setting; New York (3 regions: Queens, Bronx, Manhattan), USA	IG 22 CG 24	Not reported	13 months	Nurse managers and visiting staff nurses	Diploma IG (*n* = 0)/CG (*n* = 1) Associate degree IG (*n* = 4)/CG (*n* = 4) Bachelor’s degree IG (*n* = 10)/CG (*n* = 10) Master’s degree IG (*n* = 7)/CG (*n* = 5) Not reported IG (*n* = 1)/CG (*n* = 4)
Dizon et al.	Randomised controlled trial	Training centre at the University of Santo Tomas, Manila, Philippines	IG 27 FU 1 (post intervention) 27 FU 2 (3 month post intervention) 15 CG 27 FU 1 (post intervention) 25 FU 2 (3 month post intervention) 11	IG (median, IQR) 29 (26–36) CG (median, IQR) 28 (25–30)	3 month	Physiotherapists	Years in practice IG (median, IQR) 4.2 (2–7.75) CG (median, IQR) 3.0 (1.13-4)

*IG* Intervention group, *CG* Control group, *FU* Follow Up, *IQR* Interquartile range

### EbM training courses

The EbM training offered varied in duration from courses lasting five hours [26] up to courses with 48 h [34] of teaching.

Bennet et al. [31] and Yost et al. [36] included all five core elements of EbM training in their program: 1) ask a question that can be answered; 2) identify appropriate sources for searching relevant information and perform a systematic literature search; 3) critically appraise selected publications on key elements; 4) implement EbM in everyday clinical practice; 5) communicate trial results to patients and consumers. Meyer et al. [11], Dizon et al. [28], Lizarondo et al. [33], McCluskey et al. [34], Varnell et al. [35], Courey et al. [30] and Levin et al. [27] included the first four elements, while Stevenson et al. [26], Chen et al. [29] and Jalali-Nia et al. [25] included only the first three elements. The report by Kim et al. [32] did not provide a full description of the training’s elements.

Eight training programs [11, 25, 26, 29, 31, 33, 35, 36] offered classroom-based activities for teaching the principles of EbM. Five trials [27, 28, 30, 32, 34] included co-intervention in addition to classroom teaching, such as mentorship in participants’ homes or institutions, online support, email lists to facilitate communication or presentation of relevant literature in clinical settings.

A description of EbM training programs is provided in Table 2.

**Table 2:** Description of EbM training programs

Reference	Duration	Content	Material used	Method of delivery
Bennet et al.	13-week period (two hours per week)	Workshop: ask a clinical question; find evidence; critically appraise evidence; integrate the evidence with clinical expertise, patients values and circumstances Appraisal and implementation of clinical practice guidelines Communicating evidence to patients	Clinical examples and research articles	Didactic lectures, tutorial and workshop formats Database searching Presentation of an appraisal of a clinically relevant topic Role play of communicating research evidence to the patient
Kim et al.	6 8-h educational sessions	Theory of experimental learning, mentorship and resources for nurse leader and staff nurse	Not reported	Implementation of a clinical practice project in home institution Fellowship program culminates in a graduation ceremony and an EbM conference
Lizarondo et al.	6-monthly journal club sessions (each lasting an hour)	Workshop: asking a question, developing a search strategy, critical appraisal, evidence implementation and evaluation Discussion of methodological quality, key findings and issues pertaining to the implementation in clinical practice of one study	Articles from scientific journals, self-help kits on statistics	Discussion of one study ending with the resolution of a clinical problem and how to utilize evidence in making clinical decisions and evaluating its effects
McCluskey et al.	3 2-day workshops during one month	Lectures, practical sessions and discussion on: Process of evidence-based practice; asking a focused clinical question; searching electronic databases; critical appraisal of qualitative and quantitative research; interpreting statistics in randomized controlled trials; overcoming barriers when making the change to evidence-based practice	Not reported	Workshops with the assistance of a health librarian. Participants developed a critically appraised topic (CAT) Participants wrote a clinical question about the effectiveness of an occupational therapy intervention CATs were presented at a conference and uploaded to a website Email list to facilitate communication Reminders and individual feedback about the assignment
Meyer et al.	EbM courses over 1 to 3 days 2 courses of 8 lessons (lasting 45 min each), 2 courses of 16 lessons, and one course of 20 lessons. 7 content modules were obligatory and 5 were optional, depending on the length of the course.	Information on treatment benefit and safety provided through public media Fallacies of observational research Evidence necessary to draw conclusions about efficacy and safety of an intervention Framing of data: presenting relative risk reduction to exaggerate reception of treatment benefits Critical appraisal of a randomized controlled trial Drafting a searchable question; introduction to databases Accuracy and validity of diagnostic tests and techniques Validity of patient information brochures on diabetes	Sections from two video-taped TV features showing expert discussion Worksheet with key questions Abstract and tables of the Nurses’ Health Study; English-German vocabulary list and critical appraisal sheet PowerPoint slides displaying a fictitious observational study PowerPoint slides displaying study flow, baseline data, and results of the Women’s Health Initiative study on prevention of cardiovascular disease through hormone replacement therapy PowerPoint slides displaying an advertisement for Simvastatin Worksheet comprising information from the 4S-study, 2x2 table sheets and pocket calculators Misleading patient information sheet on hormone replacement therapy Worksheet on balanced reporting of benefit, lack of benefit and adverse effects of interventions German translation of the STOP-NIDDM study. Glossary and critical appraisal sheet Worksheets comprising general information on biomedical databases and relevant Internet addresses Worksheet on validity criteria for diagnostic tests Consumer information on the accuracy and practicability of blood pressure devices Abstract and tables of a validation study on a blood pressure self-measuring device. German patient or consumer information brochures on diabetes; German version of the DISCERN instrument	Observation and plenary discussion Presentation, individual work or work and analysis in pairs Computing relative and absolute risk, event rates etc. by 2x2 tables
Varnell et al.	8-week EbP educational program (2 h each week)	History of EbP; asking clinical questions; conducting literature searches; research designs; evaluating qualitative and quantitative research; implementing EbP change; and evaluating change in practice	Not reported	Delivery by four university faculty members with expertise in EbP Didactic presentations, group discussions, hands-on practice in writing clinical questions and conducting online literature searches Group work evaluating sample qualitative and quantitative research articles
Yost et al.	5-day workshop (4 h in large group sessions, 18 h in small group sessions)	Large group sessions related to EIDM (evidence informed decision making) Small group sessions focused on searching for, accessing and critical appraisal of the evidence Each small group conducted critical appraisal of therapy; intervention studies; systematic reviews; meta-analyses and practice guidelines	Background reading and studies used to practice critical appraisal techniques	Large and small group sessions, individual study time and opportunities to work with a trained librarian Participants received reading materials in advance of the worksh
Chen et al.	32-h course	Literature search Critical reading of articles	Guidelines on how to read and analyse articles	Teachers: two experienced instructors who designed the course Students practiced three report critiques and presented their critiques orally Assignments: Completing a reference list of a literature search written in APA format Presenting a literature critique of current nursing journal article Writing a literature article summary card that records the critical content and source of the article
Courey et al.	1 day workshop following weekly presentation of articles implemented into the one-semester course on Foundations of Nursing	Access and evaluate professional nursing literature	Not reported	Lecture, discussion, hands-on activities, and collaborative learning Access, evaluate, and utilize professional nursing journal articles Presenting relevant literature to peers in clinical post-conference settings
Jalali-Nia et al.	1-day workshop following weekly 2-h meetings with a tutor and the main researcher over 12 weeks	Developing a clinical question using the PICO format, searching for evidence, reading and critiquing nursing research, discussing articles, synthesizing the evidence, and developing a summary of findings	Articles for discussion	Intervention included four phases: First phase: two tutors teaching the mentors regarding the principles of the evidence-based approach to education Second phase: 1-day workshop for the intervention group provided by the primary researcher Third phase: Two medical-surgical courses were taught Fourth phase: 20 students were divided into four groups. Students met weekly for two hours Each student group prepared a paper summarizing the search process, a specific evaluation of each study and its application to practice Findings were presented and discussed in an oral presentation
Stevenson et al.	5 h of training	Evidence-based principles including the use of opinion leaders Aspects of EbP including low back pain management Critical appraisal skills and literature searching skills	Not reported	Presentation in a relaxed and open format Learning strategies included: teaching, discussion, reflective thinking, active experimentation and peer group teaching
Levin et al.	Intervention phase lasting 16 weeks 4-week period consisting of four 1-h classes, followed by an EbP mentor on 1 day a week for 2 h over a period of 12 weeks	Definition of EbP and rationale for use in clinical decision making Developing focused, searchable clinical questions; finding the evidence Basic concepts of a systematic review, specifically reading and critically appraising a meta-analysis	EbP toolkit which included narrative text on the content of the presentations Environmental prompts (e.g. posters that encourage the nurses to use EbP)	Session delivered by experts in the field EbP mentor met with nurses to facilitate their work and serve as an informal teacher of how to implement EbP concepts Sessions were prescheduled for 1 and 1.5 h each week
Dizon et al.	1 day workshop with follow-up online support	Workshop with following contents: introduction to EbM, hierarchy of evidence and study designs, drafting the clinical question using the PICO format, designing the search, critical appraisal of the evidence, answering the clinical question based from the evidence found	EbM Checklist, online EbM support, printed materials	Training program was modeled with fixed/constant and variable components Fixed components: one day face-to-face training with lectures, practical sessions Variable components: online EbM training package, EbM checklist to assist participants to apply the evidence in practice

### Risk of bias in included before-after trials

Details of risk of bias in the included before-after trials are displayed in Table 3.

**Table 3:**
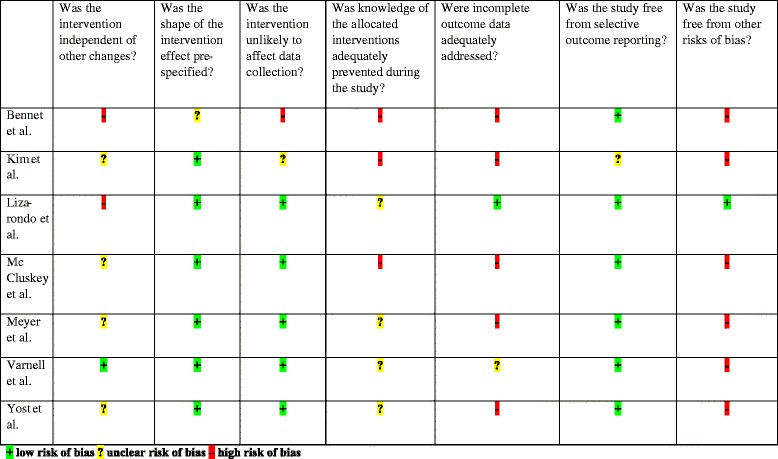
Risk of bias summary for the included before-after trials

The trials by Meyer et al. [11], Varnell et al. [35] and Lizarondo et al. [33] had the lowest risk of bias, while Yost et al. [36], Kim et al. [32] and Bennet et al. [31] had the highest risk of bias.

### Risk of bias in included controlled and randomised controlled trials

Details of risk of bias in the included controlled and randomised controlled trials are displayed in Table 4. From the included controlled trials, the trial by Courey et al. [30] had a higher risk of bias than the trial by Chen et al. [29]. From the included randomised controlled trials, the trials by Dizon et al. [28] and Levin et al. [27] had the lowest risk of bias, while the trial by Stevenson et al. [26] had the highest risk of bias.

**Table 4:**
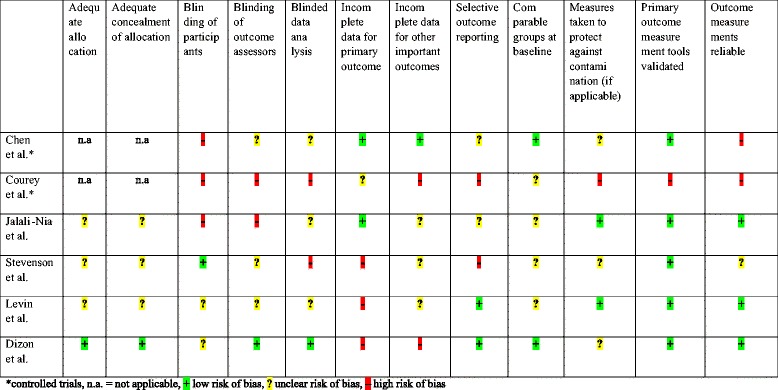
Risk of bias summary for the included controlled and randomized controlled trials

### Intervention effects

Details of intervention effects and applied assessment instruments are displayed in Additional file 1.

### Impact on attitudes

Two before-after trials [31, 32] reported that attitudes towards evidence-based practice did not significantly improve after EbM training, while one trial [35] reported higher scores on the attitude scale at the end of the program. Lizarondo et al. [33] examined the impact of EbM training on the attitudes of different associated healthcare disciplines. A significant improvement in attitudes was seen only for physiotherapists, but not for speech pathologists, occupational therapists, social workers or dieticians/nutritionists.

The controlled clinical trial by Courey et al. [30] reported a decrease in positive attitude from pre- to post-test in the intervention group and no change in the control group.

In a randomised controlled trial, Jalali-Nia et al. [25] showed a statistically significant difference between the intervention and the control group with higher scores for positive attitudes in the intervention group. Dizon et al. [28] demonstrated significantly increased positive attitudes in the intervention group immediately post-training and three month post-training. The results obtained by Stevenson et al. [26] also demonstrated small effects on positive attitudes towards the EbM concept. However, the reporting in the original paper was somehow inconclusive and a request to the authors remained unsuccessful. The specific affected element remains unclear. In the randomised controlled trial by Levin et al. [27] a statistically significant improvement in the intervention group compared with the control group was seen twice: first after the 16-week educational and mentored intervention period and secondly (nine months later) after an evidence-based practice (EbP) implementation project.

### Impact on knowledge and skills

Five before-after trials tested whether EbM training leads to an increase in EbM-related knowledge. Three trials additionally tested whether knowledge in EbM is influenced by training. Bennet et al. [31], Meyer et al. [11], and Lizarondo et al. [33] documented that participation in the course was associated with a significant increase in knowledge directly after the training. Meyer et al. [11] also observed increased skills in EbM. McCluskey et al. [34] collected data at baseline, post-training and eight months later. There was a significant increase in knowledge when baseline and post-workshop scores were compared. Improved knowledge scores were maintained after eight months. Yost et al. [36] collected data at baseline, post-training and six months later. Knowledge and skills increased significantly from baseline to post-training measurement and from baseline to six-month follow-up. The post-training measurement compared to that at six months follow-up showed a significant decrease in knowledge and skills. In their controlled clinical trial, Chen et al. [29] showed significantly increased scores for knowledge and skills in the intervention and in the control group. However, the mean score in the intervention group was significantly higher than the mean score of the control group. The randomised controlled trial by Dizon et al. [28] found significantly increased scores for knowledge and skills in the intervention group compared with a waitlist control group directly post-training and at three months follow up.

### Impact on EbM implementation and patient-relevant outcomes

Three before-after trials and two randomised controlled trials tested whether EbM training leads to an increased EbM implementation. Lizarondo et al. [33] tested the impact of EbM training on different associated healthcare disciplines. Physical therapists, social workers and dieticians/nutritionists showed statistically significant, positive changes in EbM implementation scores but speech pathologists and occupational therapists did not. While Varnell et al. [35] also reported higher scores on EbM implementation scales at the end of the program, Yost et al. [36] found no significant increase in EbM implementation behaviours from baseline to six-month follow-up. Levin et al. [27] demonstrated in a randomised controlled design more EbM implementation behaviours in the intervention group than in the control group at the end of the training. In the randomised controlled trial by Dizon et al. [28] improved EbM implementation behaviours were seen in the intervention group but not in the control group.

None of the trials assessed patient-relevant outcomes.

### Reporting quality with regard to the development and implementation of a complex intervention

A total of eleven trials described the elements of the applied training in detail. However, reporting quality varied regarding the description of the interventions’ development. Most of the trials provided insufficient information concerning piloting, description of the implementation strategy and the methods of assessing participants’ preferences, interests and experiences. Almost no information was provided on process evaluation and costs or resources needed for implementation. Details of the reporting quality are shown in Table 5. In summary, the trials by Dizon et al. [28], Levin et al. [27], Varnell et al. [35] and Meyer et al. [11] showed the highest quality of reporting, while Jalali-Nia et al. [25], Chen et al. [29] and Courey et al. [30] demonstrated poor reporting quality on the development and piloting of the complex intervention.

**Table 5:** Reporting quality with regard to the development and evaluation of a complex intervention

	Bennet et al.	Kim et al.	Lizarondo et al.	McCluskey et al.	Meyer et al.	Varnell et al.	Yost et al.	Chen et al.	Courey et al.	Jalali-NIa et al.	Stevenson et al.	Levin et al.	Dizon et al.
Description and delivery of the intervention													
ᅟDescription of all components of the intervention	+	-	+	+	+	+	+	+	+	+	+	+	+
ᅟDescription of the control intervention (comparator)	n. a.	n. a.	n. a.	n. a.	n. a.	n. a.	n. a.	-	-	-	+	+	+
Description of the intervention’s development													
ᅟDescription of the intervention’s underlying theoretical considerations	+	-	+	+	+	+	-	+	-	-	+	+	+
ᅟRationale for the selection of the intervention’s components	-	-	+	+	+	-	+	-	-	-	-	+	+
ᅟIllustration of any intended interactions between different components	-	-	-	-	-	+	-	-	-	-	-	+	+
ᅟRationale for the aim/essential functions of the intervention’s components, including the evidence whether the components are appropriate for achieving this goal	+	-	-	+	+	+	-	-	-	-	-	-	+
ᅟConsideration of contextual factors and determinants of the setting in the modeling of the intervention	+	-	+	-	+	-	+	-	+	-	-	+	+
Information on a pilot-test													
ᅟInformation on pilot-testing	n. a.	-	+	n. a.	n. a.	-	-	-	-	-	-	+	+
ᅟIn case of pilot-test: presentation of all relevant results and their impact on the modeling of the final intervention	n. a.	n. a.	-	n. a.	n. a.	n. a.	n. a.	n. a.	n. a.	n. a.	n. a.	+	+
Description of the implementation strategy													
ᅟIf the study was conducted in different clusters or centers: description of a standardised implementation strategy throughout the centers	n. a.	+	-	-	n. a.	+	-	-	n. a.	-	-	-	n. a.
Methods of assessing participant’s preferences, interests, experiences													
ᅟDescription of facilitators or barriers revealed by the process evaluation which have influenced the interventions’ implementation	-	+	-	-	-	+	-	-	-	-	-	-	+
Description of a process evaluation													
ᅟDescription of an evaluation of the implementation process	-	-	-	-	-	-	-	-	-	-	-	-	-
ᅟDescription of unexpected interactions between components of the intervention and the environment in which the intervention was implemented	-	+	-	-	-	-	-	-	-	-	-	-	-
Information on costs, resources needed for the intervention’s implementation													
ᅟDescription of costs or required resources for the intervention’s implementation	-	-	-	-	-	-	-	-	-	-	-	-	-

## Discussion

The results of this systematic review on the effectiveness of EbM training show some impact on the knowledge and skills of healthcare professionals. However, the improvement in knowledge and skills was often rather small. Three trials demonstrated significant higher scores on EbM implementation scales and one trial reported improved EbM implementation behaviours measured by activity diaries. However, improvement of EbM implementation was self-perceived; hence, the impact on the practical application of EbM remains unknown.

Adequate knowledge and skills are indispensable for successful implementation of EbM but are not the only prerequisite. Negative attitudes, low management priority and no willingness to change current practice models are well-described barriers of EbM implementation [37].

Conflicting results were seen regarding the impact of EbM training on attitudes towards EbM. While some trials reported improvement, other trials did not. One trial even reported a decrease in positive attitude from pre- to post-intervention in the EbM training group. The trial by Lizarondo et al. [33] demonstrates inconsistent outcomes across different disciplines of healthcare professionals following EbM training. Some disciplines showed statistically significant improvements in all outcomes; others did not. It is unclear whether the training programs evaluated in this review will be comparably effective across all branches of healthcare professionals. Current systematic reviews also conclude that teaching interventions may positively influence EbM-related knowledge, skills and attitudes in healthcare professionals [17, 38].

These reviews did not make any attempt to take the nature of complex interventions into account, neither did they refer even to suggested models like the UK MRC framework.

EbM training for healthcare professionals as complex interventions comprise different elements that act interdependently, e.g. train-the-trainer modules, number of sessions, curriculum, corresponding media and materials. There are also different contextual factors, such as setting and didactic strategies, as well as the educational and professional background of the participants that may influence the intervention effects.

The majority of the trials offered exclusively classroom-based EbM training, while some trials included co-intervention in addition to classroom teaching. There is much debate about what is the best type of educational activity to achieve a substantial increase in putting EbM into practice [39]. Empirical evidence exists in favour of clinically integrated EbM training over classroom-based teaching in relation to changing behaviour [8, 16, 40] which might be an indicator for successful implementation.

The process of developing a complex intervention has several phases. Not all research will need to begin at the beginning and work stepwise through an entire framework for example provided by the MRC. Sometimes evidence already exists; sometimes steps are more or less important [18]. However, complete reporting on the development and piloting of all components is most important in order to interpret the outcomes of a complex intervention. The trials included in our review provided insufficient reports on the development and implementation of the EbM training. Piloting was rarely reported by the randomised controlled trials. However, avoiding proper piloting might lead to non-effective interventions since no attempt was made to understand and reduce procedural, clinical, and methodological uncertainties in advance of the implementation of the intervention within the main trial. There was also insufficient information provided about participants’ preferences, interests and experiences. This might result in low acceptability of the intervention. Unfortunately, almost no information was provided about process evaluation and the costs or resources required. Thus, no insight was given into why an EbM training might have unexpected outcomes, why a successful training worked or how it might be optimized. Information about costs and resources is important for decision-makers.

Our review has several strengths. In order to increase the validity of the results only trials using validated assessment instruments have been included. Our review is the first taking the complexity of the included interventions into account. Therefore, we applied a specific criteria list on the quality of reporting of complex interventions [23]. Reporting a complex intervention trial according to the requirements of CReDECI might improve transparency and understanding of the intervention and might also have an impact on the value of future systematic reviews dealing with complex interventions

A full description of the EbM training and an understanding of its elements are crucial for the reproduction of the intervention’s evaluation, the adaption of EbM training to different settings, and for long-term implementation [41, 42].

Our review has some limitations. Of the seven contacted authors, only two replied to our request. Since we considered only English or German language publications for inclusion, a language bias could not be ruled out.

## Conclusions

There is insufficient evidence that available EbM trainings for healthcare professionals are likely to result in a meaningful change in EbM behaviour. Future trials should not focus only on participants’ knowledge, attitudes and skills. EbM training is also supposed to foster healthcare professionals’ use of EbM. Relevant outcomes should be used to assess the effectiveness of EbM training and to investigate whether such courses lead to changes in care processes or patient-relevant outcomes.

In this review, conclusions about effective training elements cannot be made due to the poor reporting quality of the included trials. In order to generate formats suitable for long-term implementation, future EbM training should be carefully developed, theory-based, piloted and finally investigated in a robust, randomised controlled trial.

## Additional file

Additional file 1: Table S1. Effects of the interventions. (PDF 449 kb)

## Abbreviations

EbM: Evidence-based medicine
MRC: Medical Research Council
RCTs: Randomised controlled trials
CCTs: Controlled clinical trials
MeSH: Medical Subject Headings
CONSORT: Consolidated Standards of Reporting Trials
CReDECI: Criteria for Reporting the Development and Evaluation of Complex Interventions in healthcare
EbP: Evidence-based practice
