# Supplementary material for: Effectiveness of training in evidence-based medicine skills for healthcare professionals: a systematic review
Source: BMC Med Educ. 2016 Apr 4;16:103. doi: 10.1186/s12909-016-0616-2 (PMC4820973; doi:10.1186/s12909-016-0616-2)
Supplement: Additional file 1: Table S1. — Effects of the interventions. (PDF 449 kb) [file 12909_2016_616_MOESM1_ESM.pdf]

Additional file 1: Table S1: Effects of the interventions

<table>
<tr><th>Refe-
rence</th><th>Outcomes</th><th>Tools</th><th>Results</th></tr>
<tr><td>Bennet et al.</td><td>1. Attitudes

2. Knowledge</td><td>1.
5-point Likert scale (1 = ‘strongly disagree’ and 5 = ‘strongly agree’) level of agreement with six statements regarding evidence-based practice (range 6 to 30)

2.
3 possible responses (‘agree’, ‘don’t know’ or disagree’) agreement with five statements about evidence-based practice. Items answered incorrectly or with ‘don’t know’ scored 0, a correct response scored 2 (range 0 to 10)</td><td>1.
<table><tr><th>Pre-mean score (SD)</th><th>Post-mean score (SD)</th><th>Change between pre- and post scores Mean (SD)</th><th>95% CI</th><th>p-value</th></tr><tr><td>19.80 (2.01)</td><td>20.02 (1.99)</td><td>0.22 (2.85)</td><td>(-0.96, 0.52)</td><td>0.56</td></tr></table>
2.
<table><tr><th>Pre-mean score (SD)</th><th>Post-mean score (SD)</th><th>Change between pre- and post scores Mean (SD)</th><th>95% CI</th><th>p-value</th></tr><tr><td>4.14 (2.37)</td><td>7.69 (2.31)</td><td>3.56 (2.81)</td><td>(2.83, 4.29)</td><td>< 0.001</td></tr></table></td></tr>
<tr><td>Kim et al.</td><td>Attitudes</td><td>Evidence-based Practice Questionnaire (EBPQ) by Upton and Upton: subscale attitude</td><td><table><tr><th>Pre-test mean (SEM)</th><th>Post-test mean (SEM)</th><th>Mean differences</th><th>p-value</th></tr><tr><td>5.60 (0.09)</td><td>5.75 (0.10)</td><td>0.16</td><td>0.198</td></tr></table></td></tr>
</table>

<table>
<tr>
<td>Liza-
rondo
et al.</td>
<td>1. Knowledge

2. Attitudes

3. EBP uptake</td>
<td>1.

Adapted Fresno Test (AFT)

2. + 3.

Evidence-based Practice Questionnaire (EBPQ) by Upton and Upton</td>
<td>Speech Pathology
<table>
<tr><td></td><td>Pre-test Score

Mean ±SD</td><td>Post-test Score

Mean ±SD</td><td>Mean Percentage Change

(95% CI)</td></tr>
<tr><td>Knowledge</td><td>25.30 ± 11.94</td><td>53.80 ± 23.08</td><td>134.36 (54.80 – 213.82)*</td></tr>
<tr><td>Attitude</td><td>22.50 ± 1.84</td><td>23.00 ± 2.11</td><td>2.65 (-4.16 – 9.41)</td></tr>
<tr><td>EBP Uptake</td><td>26.20 ± 8.93</td><td>32.00 ± 6.41</td><td>42.30 (-4.76 – 89.41)</td></tr>
</table>
Physiotherapy
<table>
<tr><td></td><td>Pre-test Score

Mean ±SD</td><td>Post-test Score

Mean ±SD</td><td>Mean Percentage Change

(95% CI)</td></tr>
<tr><td>Knowledge</td><td>32.26 ± 14.65</td><td>79.52 ± 18.70</td><td>245.90 (110.65 – 381.23)*</td></tr>
<tr><td>Attitude</td><td>19.50 ± 3.47</td><td>21.89 ± 4.46</td><td>15.85 (6.54 – 25.26)*</td></tr>
<tr><td>EBP Uptake</td><td>18.89 ± 6.66</td><td>26.57 ± 8.12</td><td>71.06 (12.24 – 129.88)*</td></tr>
</table>
Social Work
<table>
<tr><td></td><td>Pre-test Score

Mean ±SD</td><td>Post-test Score

Mean ±SD</td><td>Mean Percentage Change

(95% CI)</td></tr>
<tr><td>Knowledge</td><td>24.19 ± 9.87</td><td>43.06 ± 14.82</td><td>141.20 (24.09 – 258.34)*</td></tr>
</table>
</td>
</tr>
</table>

	Pre-test Score Mean ±SD	Post-test Score Mean ±SD	Mean Percentage Change (95% CI)
Attitude	19.56 ± 3.37	20.81 ± 3.23	8.04 (-0.29 – 16.39)
EBP Uptake	22.56 ± 8.14	26.00 ± 5.49	28.25 (4.73 – 51.77)*

Occupational Therapy

	Pre-test Score Mean ±SD	Post-test Score Mean ±SD	Mean Percentage Change (95% CI)
Knowledge	22.75 ± 10.87	55.58 ± 22.66	198.50 (135.71 – 261.34)*
Attitude	21.31 ± 3.13	21.75 ± 3.69	2.73 (-1.98 – 7.46)
EBP Uptake	21.56 ± 7.92	22.50 ± 8.28	16.52 (-5.86 – 38.92)

Dietetics / Nutrition

	Pre-test Score Mean ±SD	Post-test Score Mean ±SD	Mean Percentage Change (95% CI)
Knowledge	31.75 ± 9.80	59.08 ± 22.63	87.81 (50.73 – 124.93)*
Attitude	21.17 ± 3.16	21.00 ± 3.38	0.20 (-8.34 – 8.75)
EBP Uptake	18.92 ± 8.08	23.58 ± 7.22	39.18 (8.56 – 69.78)*

* Significant at 0.05

<table>
<tr>
<td>Mc Cluskey et al.</td>
<td>Knowledge</td>
<td>Adapted Fresno Test (AFT)</td>
<td>1. AFT total scores (means, SD)
<table>
<tr><td></td><td>Pre-Workshop
(n=114)</td><td>Post-Workshop
(n=106)</td><td>8-month Follow-up
(n=51)</td></tr>
<tr><td>Total score (0 to 156)</td><td>57.1 (±26.7)</td><td>78.3 (±18.6)</td><td>82.2 (±24.5)</td></tr>
</table>
AFT scores, mean differences
<table>
<tr><td></td><td>Mean Diff</td><td>95% CI</td><td>p-value</td></tr>
<tr><td>Pre- to Post-Workshop
Total score (0-156)</td><td>20.6</td><td>15.6 – 25.5</td><td>0.0001</td></tr>
<tr><td>Post-Workshop to 8-month Follow-up
Total score (0-156)</td><td>1.2</td><td>-6.0 – 8.5</td><td>0.734</td></tr>
<tr><td>Pre-Workshop to 8-month Follow-up
Total score (0-156)</td><td>23.1</td><td>14.7 – 31.6</td><td>0.0001</td></tr>
</table>
</td>
</tr>
</table>

<table>
<tr>
<td>Meyer et al.</td>
<td>Knowledge and Skills</td>
<td>Multiple choice test (13 items on three subscales)</td>
<td>
<table>
<tr><td>Total score (maximum)</td><td>Before test
Means (SD)</td><td>After test
Means (SD)</td><td>Difference
Means (SD)</td></tr>
<tr><td>Total (13.5)</td><td>4.6 (±1.9)</td><td>6.8 (±1.8)</td><td>2.2* (±2.1)</td></tr>
</table>
<table>
<tr><td>Subscales score (maximum)</td><td>Before test
Means (SD)</td><td>After test
Means (SD)</td><td>Difference
Means (SD)</td></tr>
<tr><td>Study characteristics (5)</td><td>1.9 (±1.1)</td><td>2.7 (±1.2)</td><td>0.8* (±1.3)</td></tr>
<tr><td>Computation (3)</td><td>2.3 (±0.9)</td><td>2.7 (±0.5)</td><td>0.4* (±1.0)</td></tr>
<tr><td>Communication (5.5)</td><td>0.4 (±0.6)</td><td>1.4 (±1.0)</td><td>1.0* (±1.2)</td></tr>
</table>
* Significant at 0.05
</td>
</tr>
<tr>
<td>Varnell et al.</td>
<td>1. Attitudes

2. Implementation of EBP</td>
<td>1. Evidence-based practice beliefs (EBPB) scale: 16 items (range 16 to 80)

2. Evidence-based practice implementation (EBPI) scale. 18-item Likert-type scale; Responses range from “0” (zero times) to “4” (>8 times). Range 0 to 72</td>
<td>
1. Scores of EBPB scale
<table>
<tr><td>Before test means (SD)</td><td>After test means (SD)</td><td>p-value</td></tr>
<tr><td>57.10 (±5.73)</td><td>63.73 (±4.50)</td><td><0.01</td></tr>
</table>
2. Scores of EBPI Scale
<table>
<tr><td>Before test means (SD)</td><td>After test means (SD)</td><td>p-value</td></tr>
<tr><td>15.29 (±13.65)</td><td>22.86 (±11.35)</td><td><0.01</td></tr>
</table>
</td>
</tr>
</table>

<table>
<tr>
<td>Yost et al.</td>
<td>1. Knowledge

2. Skills

3. Implementation/ behaviour</td>
<td>1. and 2. Multiple-choice test (18 items, range 0 to 36)

3. Evidence-based practice implementation (EBPI) scale. 18-item Likert-type scale; Responses range from “0” (zero times) to “4” (>8 times). Range 0 to 72</td>
<td>1. and 2. Total scores for knowledge and skills
<table>
<tr><td>Before test means (SD)</td><td>After test means (SD)</td><td>Mean difference</td><td>95% CI</td><td>p-value</td></tr>
<tr><td>9.5 (±3.4)</td><td>15.1 (±5.2)</td><td>5.6</td><td>3.7 to 7.4</td><td><0.001</td></tr>
</table>
<table>
<tr><td>After test means (SD)</td><td>6-month follow-up</td><td>Mean difference</td><td>95% CI</td><td>p-value</td></tr>
<tr><td>15.1 (±5.2)</td><td>13.2 (±5.1)</td><td>-1.9</td><td>-3.7 to -0.3</td><td>0.018</td></tr>
</table>
<table>
<tr><td>Before test means (SD)</td><td>6-month follow-up</td><td>Mean difference</td><td>95% CI</td><td>p-value</td></tr>
<tr><td>9.5 (±3.4)</td><td>13.2 (±5.1)</td><td>3.7</td><td>2.1 to 5.3</td><td><0.001</td></tr>
</table>
3. Total scores for EBPI scale
<table>
<tr><td>Before test means (SD)</td><td>6-month follow-up</td><td>Mean difference</td><td>95% CI</td><td>p-value</td></tr>
<tr><td>14.5 (±11.9)</td><td>16.2 (±11.3)</td><td>1.7</td><td>-3.8 to 0.3</td><td>0.095</td></tr>
</table>
</td>
</tr>
<tr>
<td>Chen et al.</td>
<td>Skills</td>
<td>Critique test including 45 criteria (range 0 – 45 points)</td>
<td>
<table>
<tr><td></td><td>Critique Pre-test (mean, SD)</td><td>Critique Post-test (mean, SD)</td><td>p-value</td></tr>
<tr><td>Intervention Group (n=94)</td><td>5.11 ±3.00</td><td>11.32 ±5.13</td><td><0.0001</td></tr>
<tr><td>Control Group (n=74)</td><td>4.39 ±2.59</td><td>6.59 ±3.89</td><td><0.0001</td></tr>
</table>
</td>
</tr>
</table>

<table>
<tr><td>Courey et al.</td><td>Attitudes</td><td>4-point Likert-Scale, scores ranging from 4 (agree) to 1 (disagree)</td><td>Mean score for attitudes
<table>
<tr><td></td><td>Pre-test (mean, SD)</td><td>Post-test (mean, SD)</td></tr>
<tr><td>Intervention Group *</td><td>3.43 ±0,39</td><td>2.82 ±0.83</td></tr>
<tr><td>Control Group</td><td>3.54 ±0.39</td><td>3.47 ±0.50</td></tr>
</table>
* Decrease in positive attitude from pre- to post-test (Wilks`Lambda F=20.140; df=1.58; p<0.0001)</td></tr>
<tr><td>Jalali-Nia et al.</td><td>Attitudes</td><td>4-point Likert-scale, 21 questions, scores ranging from 4 (totally agree) to 1 (totally disagree)</td><td>
<table>
<tr><td></td><td>Intervention group
mean (SD), n = 20</td><td>Control group
mean (SD), n = 21</td><td>p-value</td></tr>
<tr><td>Attitude</td><td>78.14 (9.1)</td><td>59.95 (6.1)</td><td>p < 0.001</td></tr>
</table></td></tr>
<tr><td>Steven-son et al.</td><td>Attitudes</td><td>1. Agreement with 7 statements. Scores were based on a 3-point Likert-scale: agree; neither agree nor disagree; disagree</td><td>The results demonstrated changes in attitudes towards some elements of the EBP concept following the intervention package including increased preference towards trust training programs for EBP and increased support given from management to undertake EBP. The size of the effect was small, partly reflecting the fact that at baseline this group already viewed EBP in a positive light. However, putting the concept into practice through research and literature searching was given a low priority by the physiotherapists.*

*No further details reported</td></tr>
</table>

<table>
<tr>
<td>Levin et al.</td>
<td>1. Attitudes

2. Implementation of EBP</td>
<td>1. 16-item EBP belief scale (5-point Likert-scale, range 16 to 80) higher scores indicating stronger beliefs about EBP

2. Evidence-based practice implementation (EBPI) scale. 18-item Likert-type scale; Responses range from “0” (zero times) to “4” (>8 times). Range 0 to 72</td>
<td>1. Main effect for group (F1.15 = 33.105, p<0.001), a quadratic main effect of time (F1.15 = 7.335, p<0.016) and a significant interaction between group and time (F3.45 = 16.342, p=0.001)

2.Overall score of the intervention group (M=29.52) on the EBPI scale was significantly higher than that of the control group (M=10.44)
<table>
<tr><td></td><td>Intervention group</td><td>Control group</td></tr>
<tr><td>Baseline</td><td>15.40</td><td>10.53</td></tr>
<tr><td>4-month follow up</td><td>41.46*</td><td>14.83*</td></tr>
<tr><td>13-month follow up</td><td>31.64*</td><td>6.17*</td></tr>
</table>
* Significant at 0.05</td>
</tr>
<tr>
<td>Dizon et al.</td>
<td>1. Knowledge/Skills

2. Attitudes

3. Implementation/ behaviour</td>
<td>1. Adapted Fresno Test (AFT)</td>
<td>
<table>
<tr><td>AFT scores (total = 0-156)</td><td>Intervention group

Median (IQR), 95% CI</td><td>Control group

Median (IQR), 95% CI</td><td>p-value</td></tr>
<tr><td>Pre training</td><td>11.0 (8.0-32.5), 9.0-21</td><td>21.0 (5.0-29.5), 7.9-26.1</td><td>0.97</td></tr>
<tr><td>Post training</td><td>68.0 (51.5-76.8), 53.0-72.0</td><td>20.0 (9.3-30.8), 11.0-30.0</td><td><0.0001*</td></tr>
</table>
</td>
</tr>
</table>

3-month post training	53.0 (39.2-71.8), 42.0-69.1	9.0 (8.0-27.5), 9.0-15.2	<0.0001*

* Significant at 0.05

2. Six Questions related to changing practice and confidence in searching literature or undertaking critical appraisal. Questions are to be answered with agree, disagree or neither

Significant difference were found between the groups immediate post-training and three month post-training in terms of two EbM activities:

More participants in the intervention group disagreed in terms of “lacking confidence in undertaking a literature search” and more participants in the intervention group agreed in terms of “confidence in undertaking critical appraisal”

No significant differences were found between the groups in terms of the remaining four EbM activities, specifically at three month post-training.

3. Measured by activity diaries. Participants of both groups were instructed to log EbM related activities in their working environment. A distinction was made for unique cases and usual cases

Comparsion of behaviour performed by the participants when faced with new or unique cases:

	Intervention group<br>n = 14	Control group<br>n = 10	P value
EbM behaviours when faced with new or unique cases			
Participants who formulated PICO	11 (78,6%)	0 (0%)	0.0001*
Participants who logged PICO	4 (28,6%)	0 (0%)	0.037*
Participants who searched research evidence	10 (71,4%)	3 (30%)	0.04*
Participants who appraised evidence	4 (28,6%)	0 (0%)	0.037*

			Participants who applied evidence	4 (28,6%)	0 (0%)	0.037*
			**Non-** EbM behaviours when faced with new or unique cases			
			Participants who asked colleagues	11 (78,6%)	8 (80%)	0.93
			Participants who asked MD	6 (42.9%)	10 (100%)	0.003*
			Participants who read textbooks	8 (57.1%)	10 (100%)	0.017*

* Significant at 0,05

Comparsion of behaviour performed by the participants when faced with usual cases:

	Intervention group<br>N = 9	Control group<br>N = 10	p-value
EbM behaviours when faced with usual cases			
Participants who formulated PICO	8 (88,9%)	0 (0%)	<0.001
Participants who logged PICO	2 (22,2%)	0 (0%)	0.16
Participants who searched research evidence	9 (100%)	3 (30%)	0.0016*
Participants who appraised evidence	3 (33.3%)	0 (0%)	0.04*
Participants who applied evidence	4 (44.4%)	0 (0%)	0.018*

			Non-EbM behaviours when faced with usual cases			
			Participants who asked colleagues	7 (77.8%)	8 (80%)	0.906
			Participants who asked MD	4 (44.4%)	7 (70%)	0.26
			Participants who read textbooks	7 (77.8%)	7 (70%)	0.70
			* Significant at 0.05			
